# Supplementary material for: Disparate patterns of movements and visits to points of interest located in urban hotspots across US metropolitan cities during COVID-19
Source: R Soc Open Sci. 2021 Jan 13;8(1):201209. doi: 10.1098/rsos.201209 (PMC7890478; doi:10.1098/rsos.201209)
Supplement: Supplementary Material [file rsos201209supp1.pdf]

SUPPLEMENTARY MATERIAL:

1. Data and code accessibility statement:

Data and relevant code for this research work are stored in GitHub: https://github.com/Qingchun-Li/COVID-Movement-Pattern-Analysis and have been archived within the Zenodo repository: https://doi.org/10.5281/zenodo.4290687.

2. Results of different clustering algorithms

Table S1 illustrates the results of silhouette coefficient of different clustering algorithms and different clustering numbers. Figure S1 illustrates the clustering results of different algorithms. We finally choose DBA (dynamic time warping barycenter averaging) with two clusters.

Table S1. Silhouette coefficients of different clustering algorithms and different clustering numbers.

	Silhouette coefficient				
Algorithms	N=2	N=3	N=4	N=5	N=6
Euclidean	0.501	0.483	0.373	0.421	0.366
DBA	0.514	0.483	0.408	0.482	0.431
Soft-DTW	0.501	0.483	0.408	0.451	0.407
K-shape	0.0006	0.01150	-0.067	0.1237	0.0778

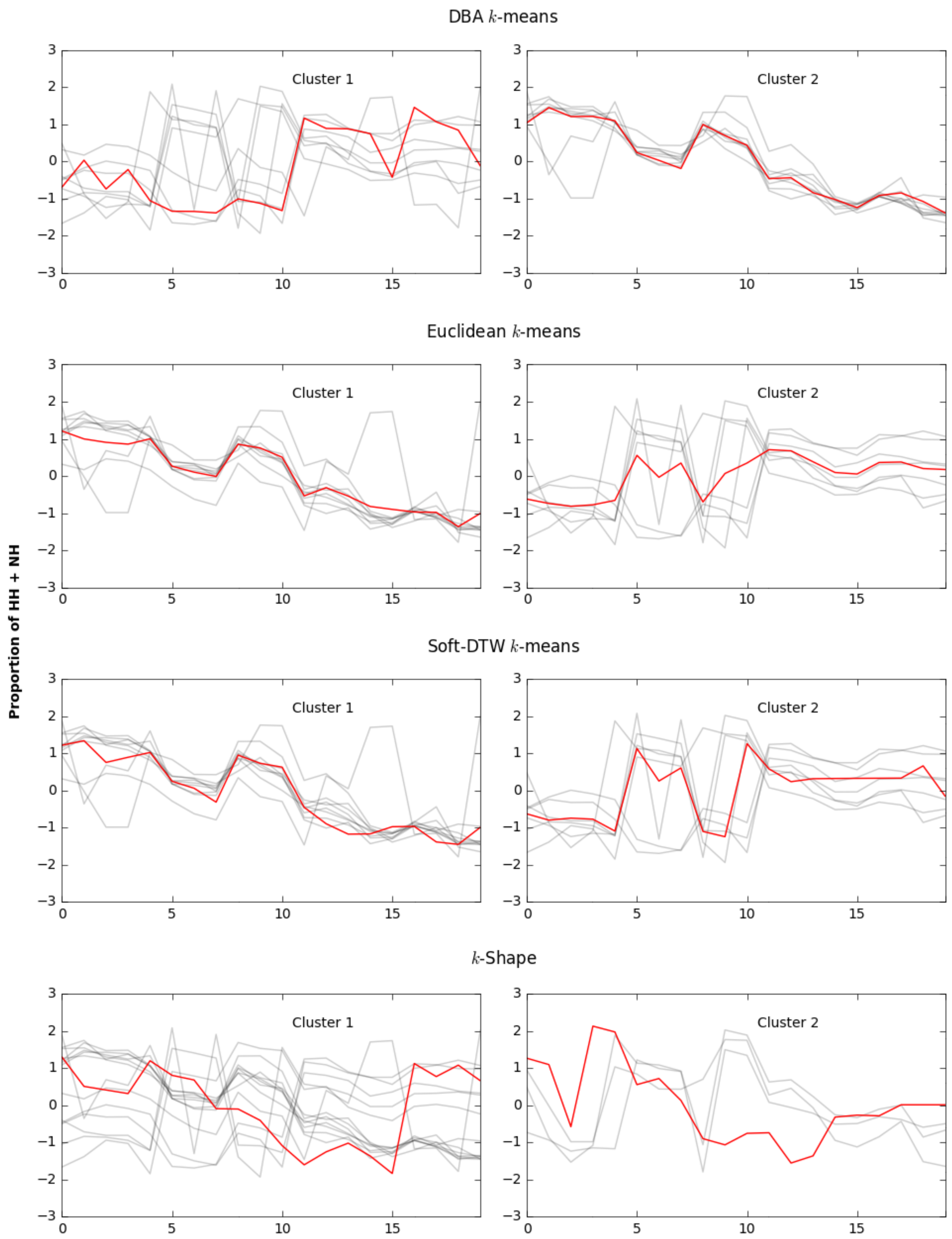

Figure S1. Results of clustering algorithms (weeks from December 30, 2019 through May 11, 2020)

3. Proof of Chi-square distribution:

For two studied moments (e.g., March 1 and March 29), weighted degree centrality of nodes are $d_{11}, d_{12}, \cdots, d_{1n}$ and $d_{21}, d_{22}, \cdots, d_{2n}$. Then the difference of weighted degree centrality of each pair of nodes are $C_1, C_2, \cdots C_n = (d_{11} - d_{21}), (d_{12} - d_{22}), \cdots, (d_{1n} - d_{2n})$. Therefore, if the weighted degree centrality of nodes in the aggregated weekly CBG-POI networks of two studied moments does not have significant changes (null hypothesis), the difference of weighted degree centrality of pair of nodes, $C_1, C_2, \cdots C_n$, approximately follows a normal distribution. The mean of the normal distribution equals to 0, and the standard deviation equals to $\sqrt{\frac{C_1^2+C_2^2+\cdots+C_n^2}{n}} = \sqrt{\overline{C^2}}$. Therefore, the $Z^2 = {C_n^2}/{\overline{C^2}}$ approximately follows a chi-square distribution with degree of freedom is 1. Osorio et al. [1] provides the result.

4. Top 7 POIs in hotspots in cities

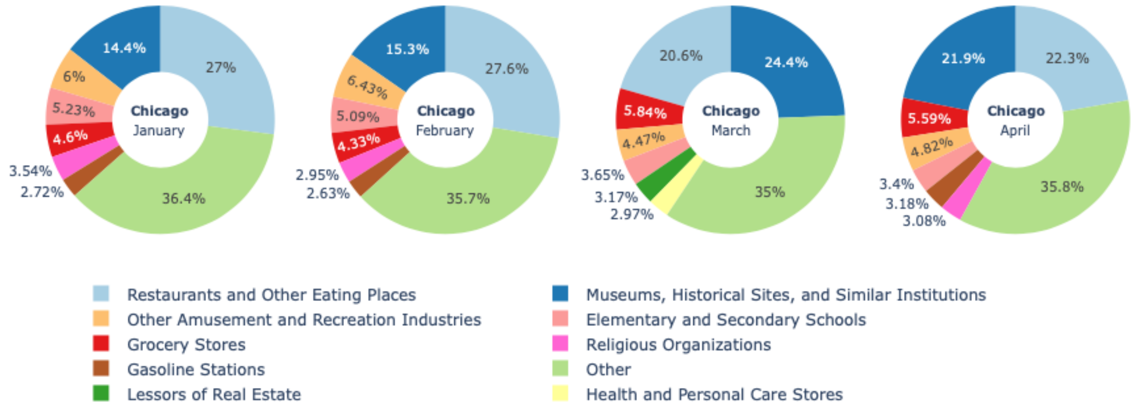

Figure S2. Chicago

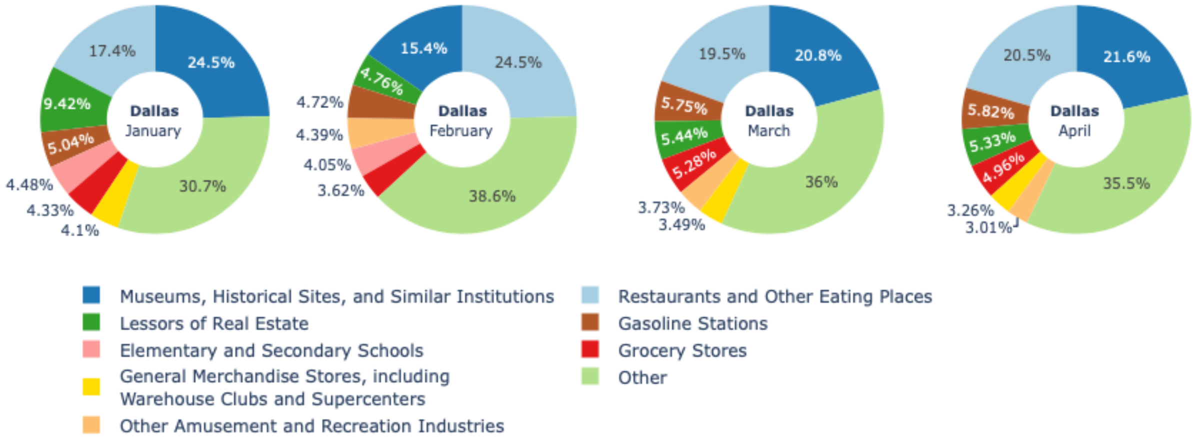

Figure S3. Dallas

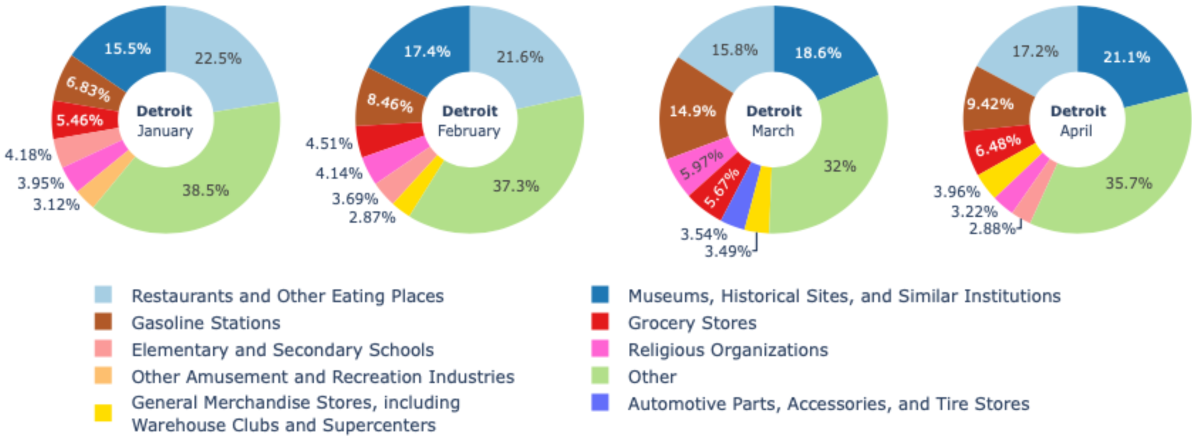

Figure S4. Detroit

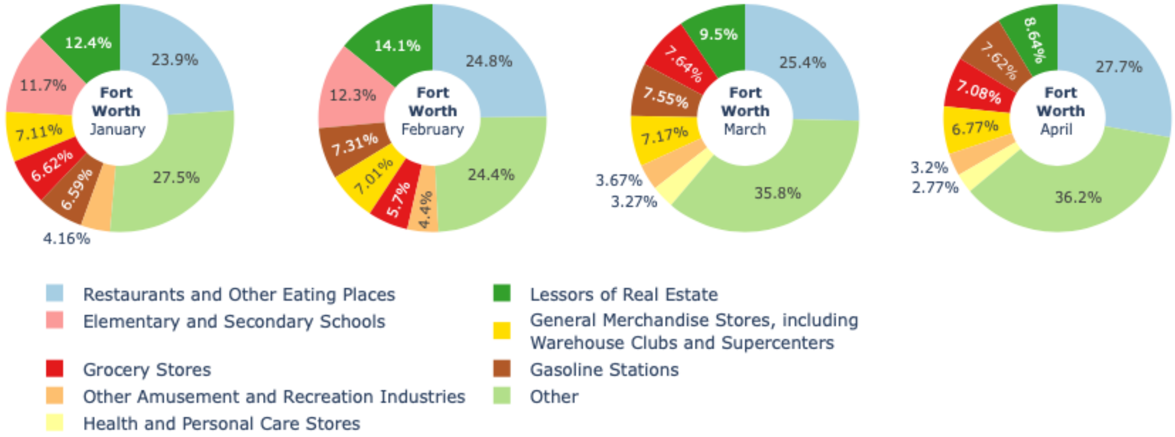

Figure S5. Fort Worth

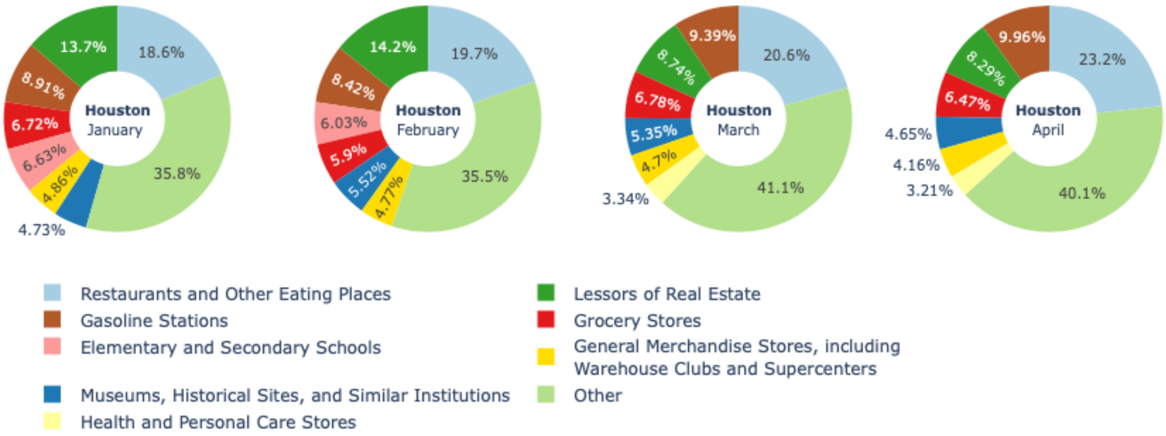

Figure S6. Houston

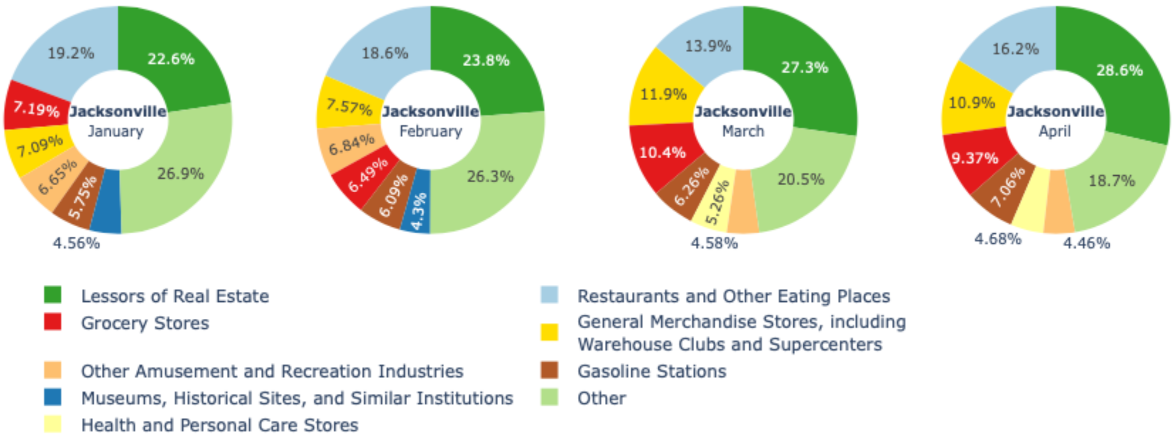

Figure S7. Jacksonville.

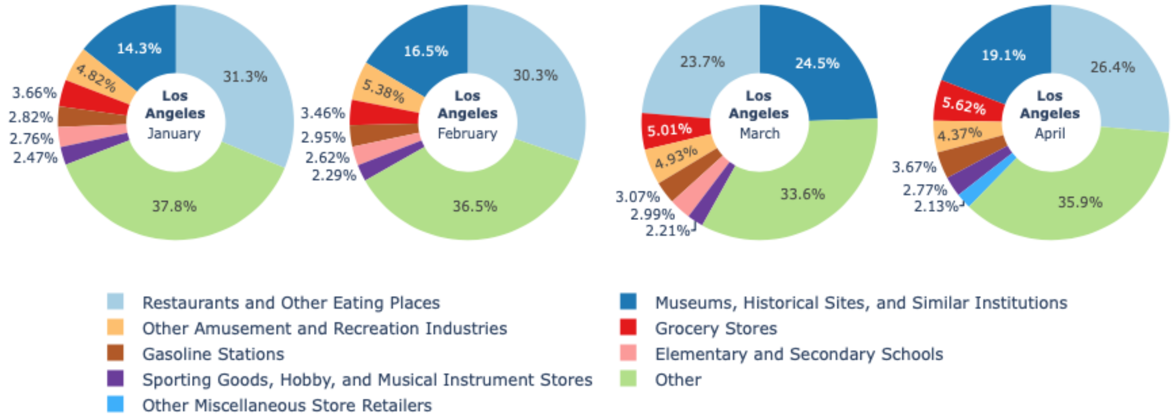

Figure S8. Los Angeles.

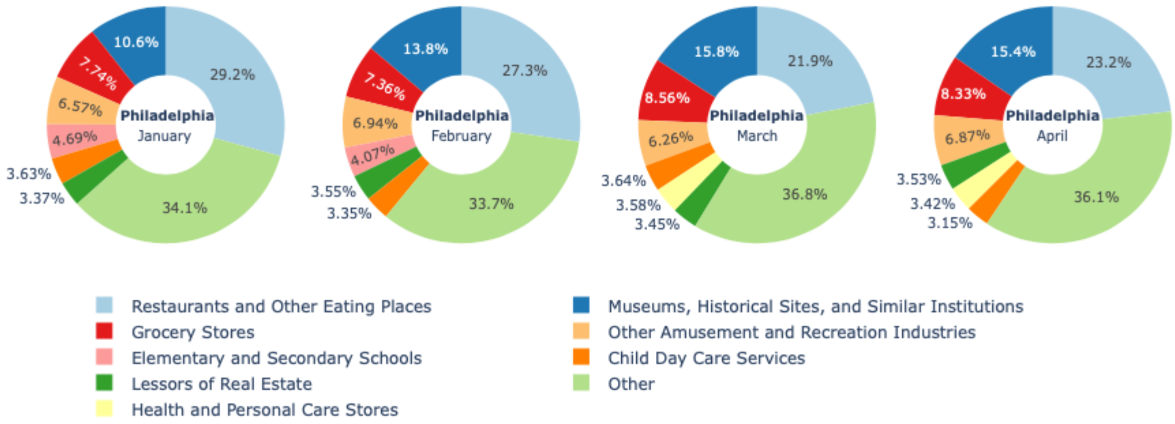

Figure S9. Philadelphia.

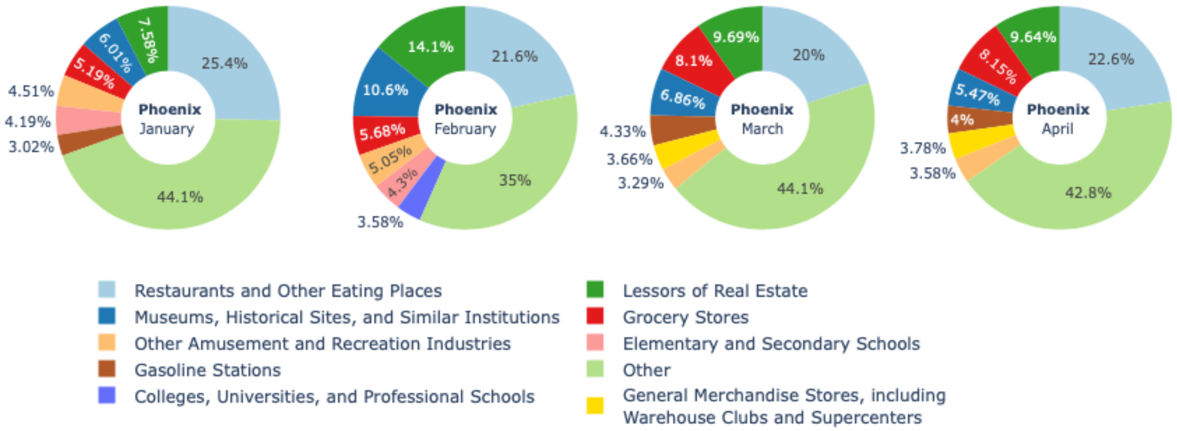

Figure S10. Phoenix.

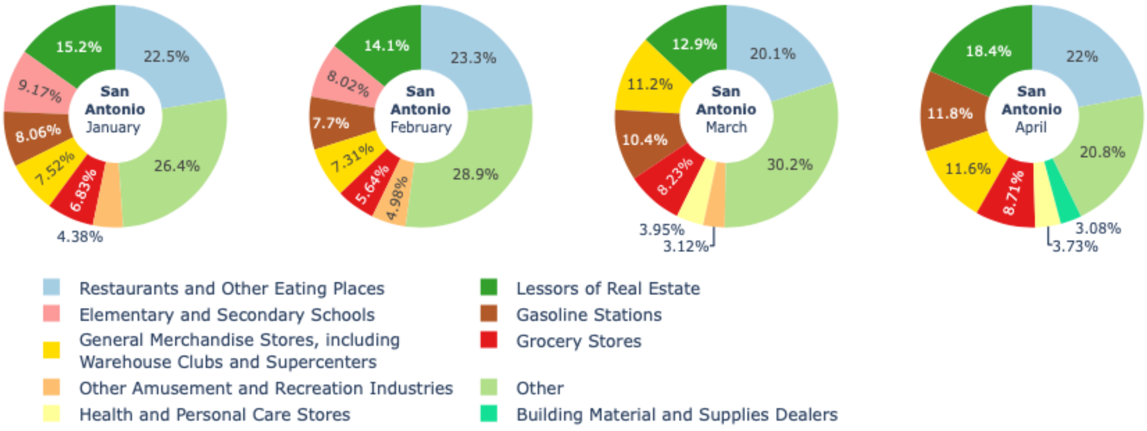

Figure S11. San Antonio.

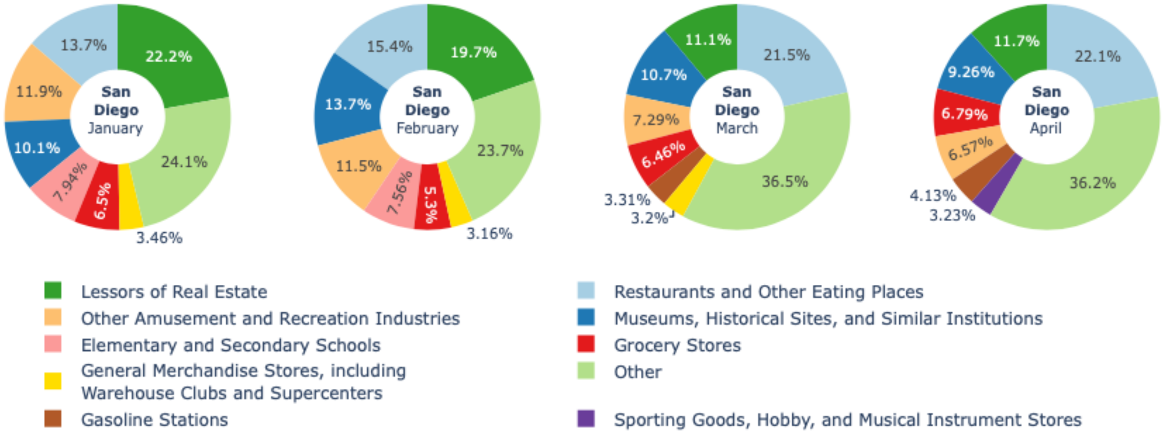

Figure S12. San Diego.

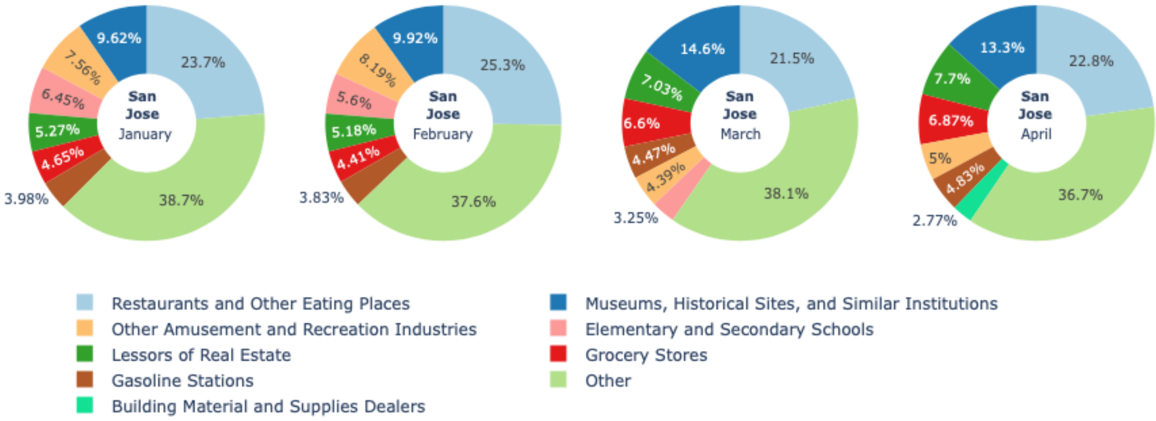

Figure S13. San Jose

5. Highly affected POIs in four studied weeks

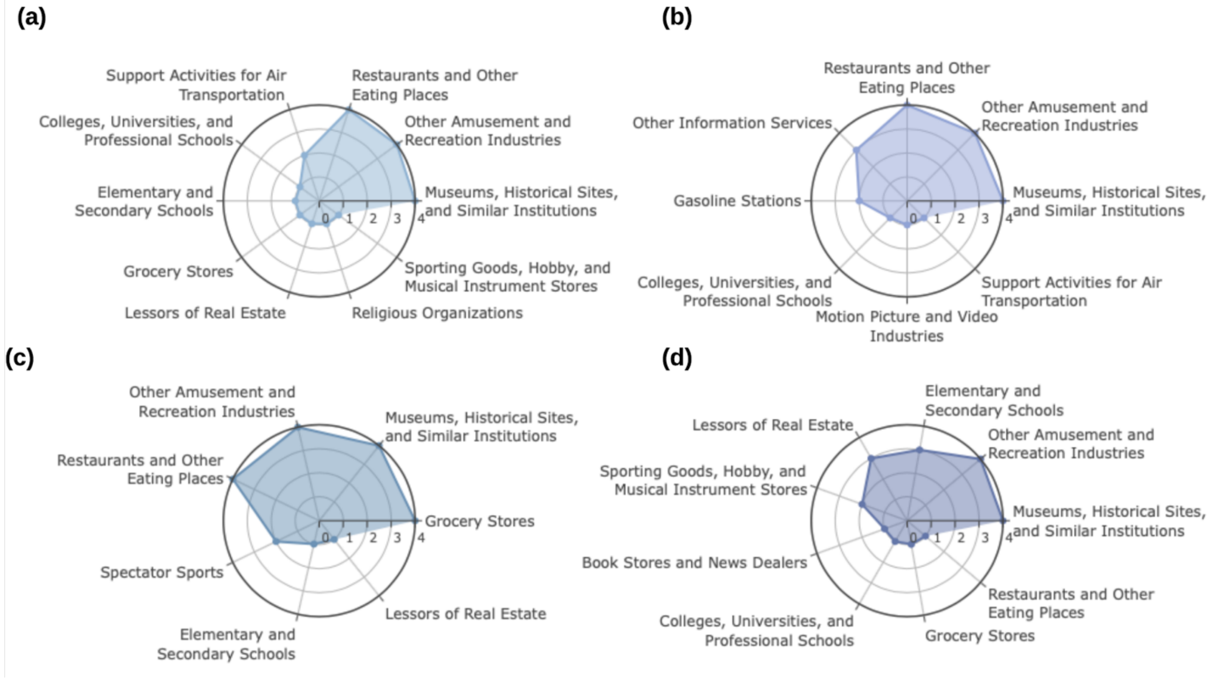

Figure S14. (a) Chicago, (b) Los Angeles, (c) Philadelphia, (d) San Jose.

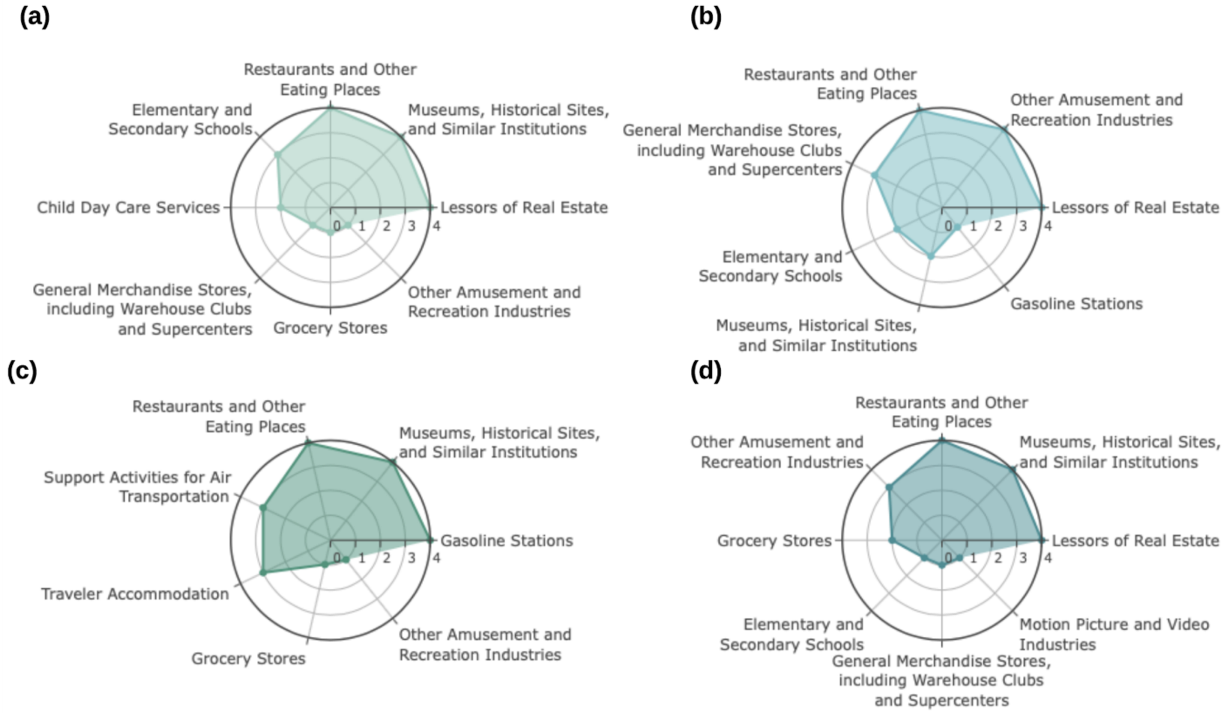

Figure S15. (a) Houston, (b) San Antonio, (c) Detroit, (d) Jacksonville.

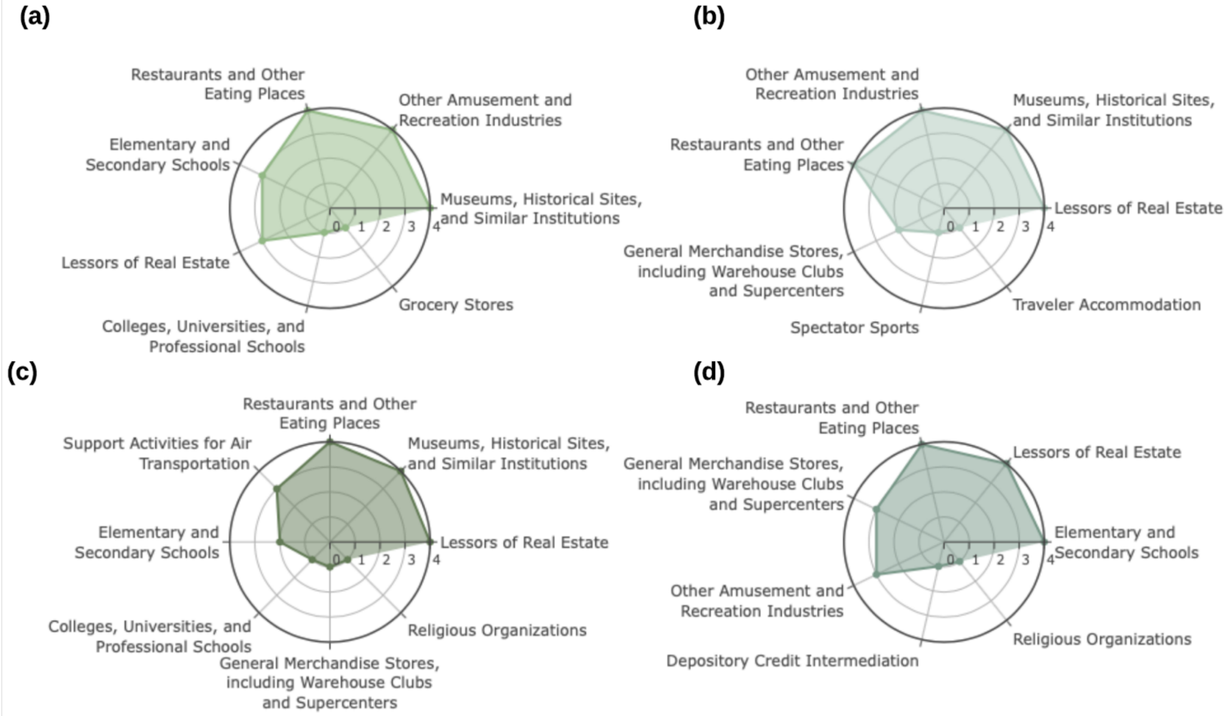

Figure S16. (a) San Diego, (b) Phoenix, (c) Dallas, (d) Fort Worth.

4. Original data of four types of movements in 16 cities:

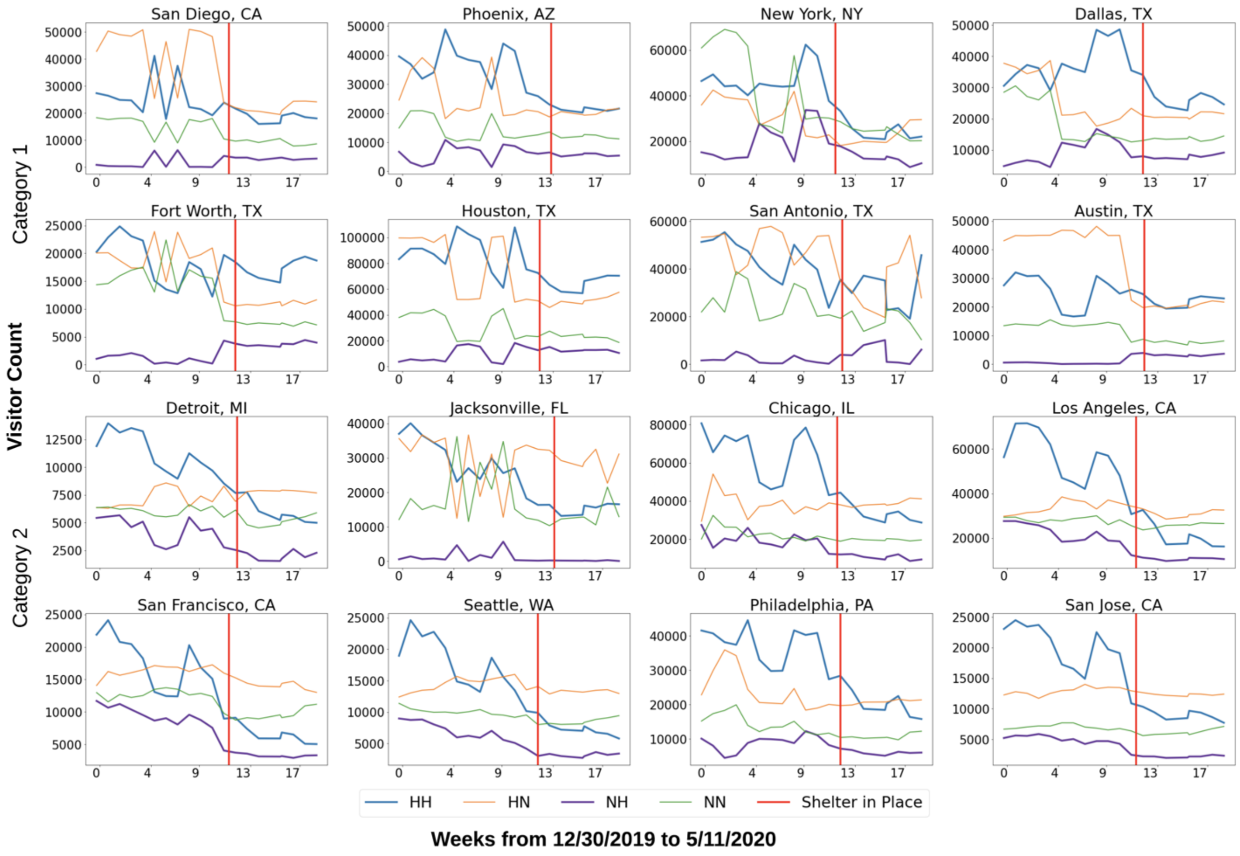

Figure S17. Visitors of four types of movements

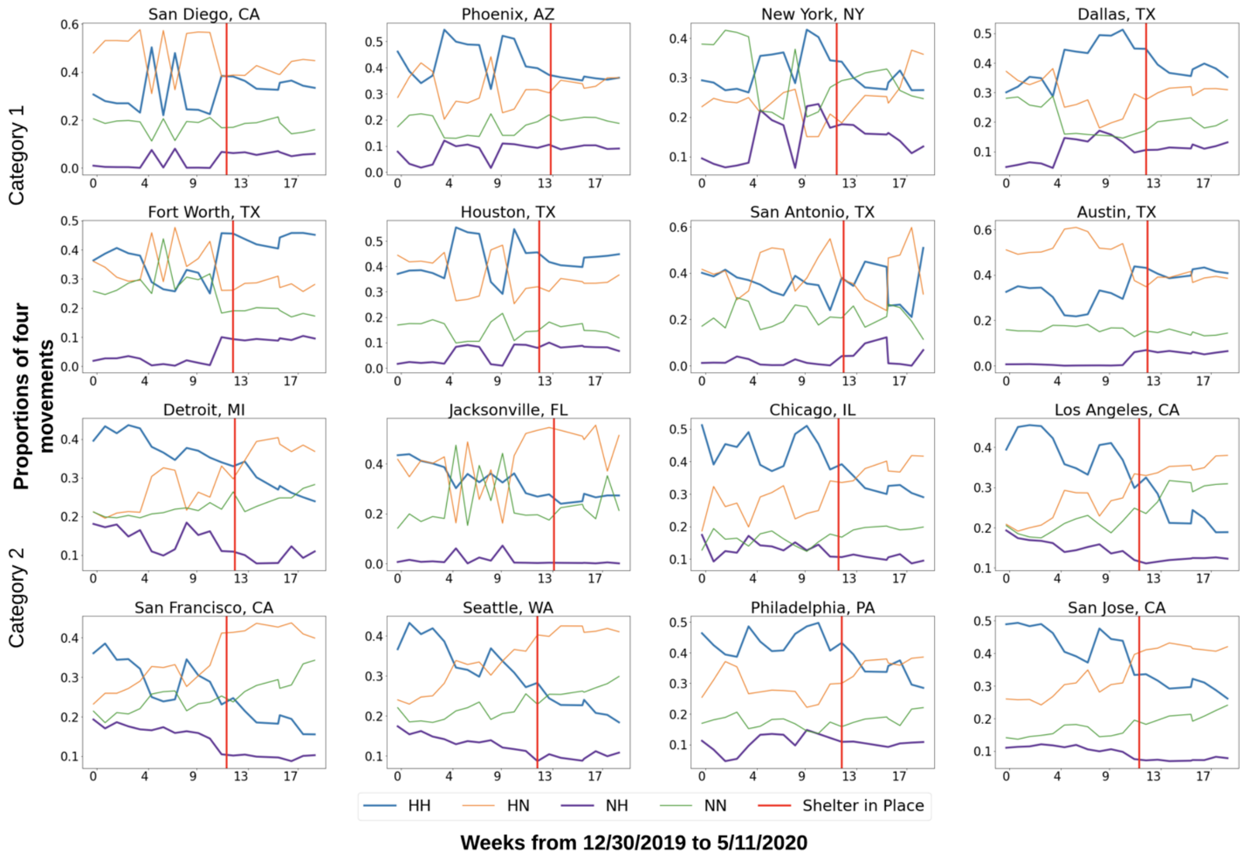

Figure S18. Proportions of four types of movements in 16 cities.

Reference in supplemental document:

1. Osorio D, Zhong Y, Li G, Huang JZ, Cai JJ. scTenifoldNet: A Machine Learning Workflow for Constructing and Comparing Transcriptome-wide Gene Regulatory Networks from Single-Cell Data. Patterns. 2020;
